# The role of neurogenic inflammation in pulp repair and the techniques used for its assessment (narrative review)

**DOI:** 10.3389/fdmed.2025.1686734

**Published:** 2025-10-09

**Authors:** Muna Sh. Ahmed, Anas F. Mahdee

**Affiliations:** Department of Restorative and Aesthetic Dentistry, College of Dentistry, University of Baghdad, Baghdad, Iraq

**Keywords:** neuropeptides, pulp innervation, regeneration, pulp response, CGRP

## Abstract

Neurogenic inflammation is pivotal in dental pulp repair, involving complex interactions between sensory nerves, immune cells, and dental pulp stem cells (DPSCs). This review aimed to identify the favorable pathways of neurogenic inflammation and neurogenic differentiation of DPSCs in the pulpal healing process. Also, to identify the techniques used to evaluate these inflammatory and differentiation processes. Both PubMed and Google Scholar databases were employed in the search strategy using keyword combinations based on MeSH terms. The search was performed for published articles in English from January 2014 to November 2024, including studies with histological and molecular findings. 29 articles only met the inclusion criteria. Neurogenic inflammation encompasses three main stages: initial (minutes to 24 h), intermediate (24 h to 3 days), and long-term response (3 days to several weeks). The immediate phase includes neuropeptide release, resulting in inflammation and recruitment of immune cells. The intermediate phase features the persistence of neuropeptides, nerve sprouting, and the initiation of repair processes. The long response phase involves resolving inflammation, angiogenesis, decreased neuropeptide levels, and neurogenesis mediated by DPSCs. Advanced methods such as IHC, RNA sequencing, electrophysiological studies, and micro-CT imaging have been employed to evaluate these mechanisms. However, limitations in real-time dynamic assessment highlight the necessity for more advanced and noninvasive procedures for direct evaluation of this complex process.

## 1 Introduction

The innervation of the dental pulp is exceedingly dense not only for perception but also for the maintenance of tooth vitality. The neurological components are mostly sensory trigeminal afferent axons with a few sympathetic efferent nerves. These sensory neurons have been shown to perform a wide range of actions in addition to their presumed sensory function, which is the reaction to the noxious stimuli affecting the tooth (1). Dental pulp reacts to injury or harmful stimuli through a complex interaction of neurogenic and immune responses, which occur over distinct temporal phases. In the immediate phase (minutes to 24 h), sensory nerves, especially C-fibers and A-δ fibers, release neuropeptides like substance P (SP) and calcitonin gene-related peptide (CGRP). This initiates vasodilation, attracts immune cells, and leads to neurogenic inflammation (2, 3). These actions are intensified by the activation of odontoblasts and the migration of dental pulp stem cells (DPSCs), which aid in early repair process (4). In the intermediate phase (24–72 h), the ongoing release of neuropeptides and nerve sprouting influence pain and tissue healing, while DPSCs further promote neurogenic differentiation (the process by which stem cells mature into neural lineages) and immune modulation. After injury, DPSCs can undergo neurogenic differentiation. This is critical for regenerating the damaged nerve fibers within the pulp, restoring sensory function (nociception), and contributing to the overall tissue repair response through the release of neurotrophic factors (5, 6). During the long-term phase (days to weeks), inflammation subsides, angiogenesis occurs, and tertiary dentin forms, all driven by neuropeptide signaling and DPSC-mediated regeneration (7, 8). To better understand the role of neurogenic inflammation and neurogenic differentiation in pulp repair, a range of advanced techniques have been employed over the last decades for evaluating both the neural and immune components of these processes (9). These methodologies enable precise detection of neuropeptides, cytokines, and immune cell markers involved in inflammation and the techniques used for stimulation and detection of neurogenic differentiation of DPSCs. Researchers have also been using a variety of lab models, from cell cultures to animal studies (10, 11), to see how neurons or their neuropeptides can modulate the pulp's inflammatory response. Animal models remain the gold standard model used by dental researchers for the majority of experimental dental studies *in vivo* (12–14). By carefully examining these techniques and models and acknowledging their limitations, proper guidance for future research can be obtained to improve our understanding of how the pulp heals and regenerates. Therefore, this review aimed to investigate the role of neurogenic inflammation and the mechanism of neurogenic differentiation of DPSCs in favorable pulp response. Also, to assess the methodology used to evaluate the neurogenic inflammatory process. This can assist in using this part of the overall pulp inflammatory process to obtain significant clinical outcomes.

## 2 Search strategy

PubMed and Google Scholar databases were used; the search strategy was based on MeSH terms in the following combinations: **(“neurogenic” OR “CGRP” OR “substance P”) AND (“pulp” OR “dental” OR “dentistry”) AND (“healing” OR “repair” OR “regeneration” OR “regenerative”).** This search was performed for articles published in English during January 2014 and November 2024. The studies included histological and molecular findings only. Case reports, narrative reviews, case series, clinical trials, social media sources, and studies with clinical and radiographic findings only were excluded. After initial research, 168 articles were identified, 52 were selected for title and abstract details, and only 29 met the inclusion criteria after full-text reading. This search strategy is well-suited for lab-based dental research that ensures systematic and efficient selection of relevant studies while promoting reproducibility and transparency of the research process. However, the reliance on MeSH terms and narrow keyword combinations may overlook studies that use different terminology.

## 3 Extracted data

The extracted data included studies, study sample, stimulative factors, testing procedure, and findings, listed in Table 1.

**Table 1:** Summary of the studies included in this review.

Studies	Study sample	Stimulative factor	Testing procedure	Findings
Chavarría-Bolaños et al., (19)	Human 1st premolars (clinical study)	Orthodontic force for 24 h	Radioimmunoassay to detect SP, CGRP, β-End, Met-Enk. - Heft-Parker visual analog scale for pain scoring.	All samples had baseline neuropeptide and endogenous opioid levels. Only SP was markedly raised after a 24 h of stimulation, and patients reported mild pain confined to the affected premolar.
Couve et al., (24)	Healthy and carious human molars (Human tissue-based clinical study).	Dental Caries	IHC to detect βIII-Tubulin and CX43 markers	Odontoblasts beneath carious lesions showed reduced Cx43 and tubulin, indicating weakened cellular junctions during inflammation.
Chmilewsky et al., (25)	- Human immature third molars (control) and others with caries (experimental) (Human tissue-based clinical study).	Dental caries	- IHC 20 μm-thick sections stained for β-III-Tubulin, and C5aR - In-Cell Western assay, to detect the same markers - ELISA test, to measure BDNF concentrations. - Neurite outgrowth assay employed Axon Investigation Systems (AXIS).	Pulp fibroblasts under carious injury express C5aR. C5a-C5aR signaling upregulates BDNF, driving neurite outgrowth and nerve sprouting, essential for dentin-pulp regeneration.
Mahdee et al., (20)	Mandibular 1st molars of Wistar rats (aged 8 weeks) (animal study)	Tooth wear	IHC for anti-α smooth muscle actin, anti-NaK-ATPase enzyme, anti-α tubulin, and anti-sodium hydrogen exchanger-1 (NHE-1).	Odontoblast processes expressed vimentin, α-actin, and NaK-ATPase to their entire lengths. α-tubulin and NHE-1. stained the complex branching near the dentino-enamel junction, acting as sensory “receptor fields” that can respond to tooth wear.
Steiner et al., (17)	Intact human third molars (control), and other inflamed teeth with irreversible pulpitis (experimental) (Human tissue-based clinical study).	Dental caries	- Radioimmunoassay (RIA)to detect secretoneurin (Nurhapsari et al.) and PE-11 markers. - Immuno-fluorescence staining for anti-SN, anti-PE-11, and anti-CGRP antibodies.	SN and PE-11 colocalized fully with CGRP nerve fibers (varicose and continuous shapes). Both peptides were associated with blood vessels, suggesting roles in neurovascular signaling.
Mahdee et al., (15)	Mesial cusps of mandibular 1st molars of male Wistar rats (animal study)	Tooth wearing	IHC to detect NGF, NGFR, CGRP, and NF	Worn regions showed faster nerve growth between 2 and 3 weeks after starting wear. Compared to intact regions, reaching a similar state after 9 weeks of trauma.
Ismiyatin et al., (18)	Maxillary molars of male Wistar rats (animal study)	Cavity preparation and dentinal applications of lipopolysaccharide.	IHC staining for anti-SP antibody.	E. coli LPS induced SP expression, peaking at 24 h and causing neurological inflammation in the dental cavity. By 72 h, SP levels declined, mirroring natural pulp inflammation.
Gallinari et al., (26)	Maxillary 1st molars of male Wistar rats (animal study)	35% H2O2-based whitening gel applied to teeth. Systemic treatment of Otosporin and paracetamol was also given.	- Histopathological Analysis with H&E Staining-IHC to localize and quantify neuropeptides SP and CGRP	Bleaching caused pulpal inflammation, necrosis, and increased CGRP and SP expressions. Otosporin treatment minimized necrosis and suppressed SP/CGRP release, while Paracetamol reduced inflammation, but had a limited impact on neuropeptide levels.
Byers, (2)	Sprague-Dawley rats molars (animal study)	Chewing forces (normal feeding against fasting)	IHC to detect and quantify PV, NFP, SYN, and CGRP nerve markers	Chewing rapidly depletes immunoreactivity for PV, NFP, SYN, and CGRP in pulp and dentin near cusp tips; this depletion is reversible during rest. No root pulp or periodontal nerve changes were observed.
da Silva et al., (3)	Male Wistar rats' maxillary molars (animal study)	38% hydrogen peroxide bleaching gel. Animals were supplied either with Ibuprofen treatment or a topical desensitizing agent (KF 2%)	- Histopathologic H&E staining. - IHC sections stained for anti-SP and anti-CGRP antibodies. detected by a light microscope.	Bleaching agent causes severe pulp inflammation that decreases over time. Ibuprofen reduces inflammation and neuropeptide expression, but it is less effective than desensitizing agents. Desensitizing agent (KF 2%) reduces inflammation and accelerates recovery.
Caviedes-Bucheli et al., (7)	Human healthy maxillary and mandibular 1st premolars (clinical study)	- Trauma from occlusion - Moderate orthodontic stresses	Radioimmunoassay (RIA) to assess levels of SP, CGRP, and VEGF.	The combined stimuli of occlusal trauma with orthodontic forces showed the greatest expression of SP, CGRP, and VEGF.
Byers and Calkins, (16)	Maxillary and mandibular molars of male Sprague-Dawley rats (animal longitudinal study)	Natural attrition (tooth wear)	- Axonal transport of ^3^H-proline to label nerve endings - Autoradiography and quantitative morphometry	The study identified six distinct dentinal nerve patterns, with only two connected to the Raschkow plexus, and over 186,600 innervated dentinal tubules in maxillary molars. Despite attrition altering nerve patterns.
Moore et al., (37)	- Primary murine pulp cells from molar and incisor teeth - hDPSCs (cell culture) - Left 1st maxillary molar of Gli1CreER;Rosa26^tdTomato^ mouse as injury model (animal study)	- CGRP and Shh ligands and inhibitors - Capsaicin-stimulated neuron-conditioned media - Dentin and pulp exposure injuries in mice	- Colony-forming unit (CFU) assays on dental cells of wild type mice for 14 days in culture media having Shh, CGRP, or both, and the units were manually counted. - qPCR for inflammatory and odontogenic markers (Shh, CGRP, and Gli1). - IHC for anti-Shh, anti-CGRP, and anti-CGRPr antibodies - Alizarin red staining for mineralization assessment	CGRP and Shh stimulate pulp cell proliferation and odontogenic differentiation, increasing inflammation and upregulating Gli1^+^ cells post-injury. Molar pulp cells show regenerative potential similar to hDPSCs. In pulp exposure injury, Gli1^+^ cells increased near intact nerve fibers while Shh and CGRP were elevated at the injury site, correlating with Hh signaling activation.
Saito et al., (8)	- Isolated odontoblasts from newborn Wistar rats' mandibular incisors - TG neurons collected from neonatal Wistar rats (cell culture)	Mechanical stimulation of trigeminal ganglion (TG) neurons - Alizarin red staining to evaluate the CGRP's effect on mineralization activity	IHC for odontoblasts and TG cells stained for anti-CALCRL, anti-CGRP, anti-DSPP, and anti-Gα_s_.	Dentin mineralization is negatively regulated by a cAMP-dependent signaling cascade that is triggered by CGRP, which is produced by mechanically activated trigeminal ganglion neurons and stimulates Gα₅-coupled receptors in odontoblasts. The inhibitory impact of CGRP on mineralization was abolished by AC inhibitors or CGRP receptor antagonists.
Nurhapsari et al., (29)	Maxillary incisors of male Wistar rats (animal study)	- Lipopolysaccharides (LPS) induce inflammation in exposed pulp tissue. - Asiatic acid (0.5%, 1%, 2%) vs. eugenol (control)	- ELISA test to determine the values of CGRP, TNF-α, beta-endorphins, MDA, and SOD. - Histopathology (H&E staining) to evaluate inflammation. - Rat Grimace Scale (RGS) for pain assessment	Asiatic acid showed significant anti-inflammatory effects, particularly at a 1% concentration, by reducing TNF-α levels and inflammatory cell infiltration. It also showed strong antioxidant activity, lowering MDA levels and increasing SOD activity. Asiatic acid also provided pain relief by elevating beta-endorphin levels and decreasing CGRP, with the 1% concentration being most effective.
Stanwick et al., (30)	Mandibular 1st molar of Osterix-Cre; Tgfbr2f/f deficient mice - Wild-type C57BL/6J mice (animal study)	Shallow cavity preparation.	- Micro-CT imaging. - Histological assessment using H&E and Masson's trichrome stains. - IHC stained for anti-CGRP antibody - In situ hybridization to confirm Sp7 gene transcripts, Tgfbr2 deletion.	Tgfbr2-deficient mice experienced delayed tertiary dentin formation and prolonged CGRP^+^ sensory nerve sprouting after injury. Sensory nerves compensated for impaired TGF-β signaling, promoting dentin repair through a neuro-pulpal crosstalk mechanism.
Erdogan et al., (27)	- Maxillary 1st molar of 7–12 weeks old calca knock out (calca^−^/^−^) and wild type mice (animal study) - Mouse pulp cell culture	Mechanical pulp exposure with bacterial inoculation	- Mouse Grimace Scale to assess pain behavior - Flow cytometry for detection of immune cell recruitment (CD45^+^, Ly6G^+^, Ly6C^+^) - IHC sections stained for anti-beta tubulin (tuj1) and anti-Ly6 g/Ly6C antibodies - Histopathological examination with H&E	CGRP contributes to spontaneous pain at day 1 post-injury, but without mechanical hypersensitivity. It also enhances neutrophil/monocyte recruitment, but did not affect tissue damage progression. CGRP depletion led to sensory afferent loss, bacterial invasion, tissue necrosis, and reduced mechanical hypersensitivity.
Martens et al., (28)	- hDPSCs isolated from extracted 3rd molars - Isolate primary Schwann cells from Sprague-Dawley rats (cell culture)	Standard culture medium containing mercaptothion (BME), trans-retinoic acid (RA), and essential fibroblast growth factor (b-FGF)	- IHC staining using anti-glial fibrillary acidic protein, anti-P75, anti-laminin, and anti-nestin antibodies - TEM for ultrastructural analysis of hDPSCs and Schwann-like cells - ELISA to measure BDNF, GDNF, neurotrophin 3 (NT-3), and b-NGF. - Neurite regeneration assay to measure the neurite length.	hDPSCs were successfully transformed into Schwann-like cells (d-hDPSCs) with upregulated expression of Schwann cell markers and reduced expression of neural progenitor markers. Besides, they self-aligned in collagen hydrogels, mimicking the “bands of Büngner” structure critical for peripheral nerve regeneration.
Cho et al., (23)	- hDPSCs CD34^+^/c-kit^+^/STRO-1^+^ sorted from primary hDPSCs, (cell culture)	- Neurogenic medium (Wang et al.) - PIN1 modulation (inhibitor juglone or Ad-PIN1 overexpression)	- Nissl staining to visualize neuronal cell bodies - RT-PCR to determine neural cells RNA - -IHC staining for anti-NeuN, GFAP, or nestin - Flow cytometric analysis, to measure anti-vesicular glutamate transporter-1 (VGluT1), anti-gamma-aminobutyric acid (GABA), and anti-tyrosine hydroxylase (TH) markers.	PIN1 participates in neuronal and glial transformation of hDPSCs. Its mRNA levels increase during neurogenic differentiation, and its inhibition with juglone suppresses neural transformation but promotes glial differentiation. Overexpression of PIN1 increases glutamatergic and GABAergic neurons among NeuN-positive hDPSCs, while decreasing dopaminergic neurons.
Heng et al., (21)	- hDPSCs obtained from the third molars - Human SCAPs and GMSCs donated from a single donor (cell culture)	Cocktail of 8 small molecules (VPA, CHIR99021, Repsox, Forskolin, SP600125, GO6983, Y-27632, Dorsomorphin)	- Morphological analysis for neural induced culture at two-time intervals (4 and 8 days). - qRT-PCR test for quantifying neural markers was done after the neural induction. - IHC staining for anti-MAP2, anti-NeuN, and anti-NSE antibodies - Western blot for assessment of anti-NSE antibody. - Fluo-4 AM calcium flux assay	Treated hDPSCs showed morphological changes, such as rounded cell bodies and neurite outgrowths, while those not treated continued to have a spindle-shaped, fibroblastic form. DPSCs and SCAPs showed upregulation of mature neural markers, while GMSCs showed upregulation of both mature and immature markers. Western blot and IHC confirmed increased protein-level expression of neuronal markers. Higher calcium transitory levels were seen in the Fluo-4 AM calcium flow test.
Li et al., (22)	DPSCs, SCAP, and GMSCs, from recently extracted human permanent teeth with gingival tissues excised (cell culture)	- Neurogenic differentiation media (neurosphere and non-neurosphere methods). - Growth factors (bFGF, EGF) - Neurogenic chemicals (forskolin, valproic acid, dbcAMP)	- RT-qPCR and IHC for Nestin, βIII-tubulin, NFM, MAP-2, synapsin, and pan-Nav markers and gene detection, respectively. Patch-clamp electrophysiology, to record K^+^ and Na + flux magnitudes outside and inside, respectively.	GMSCs showed the highest percentage (21.2%) of functional neurons capable of generating action potentials, while DPSCs had 8.3%. The neurosphere-mediated method is critical for functional neurons, producing electrophysiologically functions with voltage-gated Na^+^ and K^+^ currents and action potentials. Non-neurosphere methods failed to yield functional neurons despite morphological changes and neural marker expression.
Li et al., (34)	hDPSCs isolated from extracted human impacted 3rd molars (cell culture)	A modified neurogenic differentiation medium (IGFBP5 (overexpression or knockdown)	- RT-qPCR analysis for neural genes (NCAM and βIII-tubulin) - Western Blot analysis using neurogenic (anti-NCAM, anti-TH, anti-Nestin, and anti-βIII-Tubulin) and angiogenic markers (anti-VEGF, anti-PDGFA, anti-Angiopoietin-1). - IHC staining for anti-Nestin and anti-βIII-tubulin antibodies)	Overexpression of insulin-like growth factor binding-protein 5 (IGFBP5) upregulated neurogenic and angiogenic markers. Conversely, deletion of this protein significantly reduces these markers, impairing vascular differentiation and suppressing neurogenic differentiation, reducing neurosphere formation. DPSCs that overexpress this protein formed larger, more numerous neurosphere-like structures.
Niyazi et al., (35)	hDPSCs segregated from human impacted 3rd molars (cell culture)	Optogenetic stimulation for 90 min for 5 days	- Lentiviral transduction with CaMKIIa-hChR2(H134R) mCherry. - MTT assay for cell viability - IHC detection of anti-Nestin, anti-DCX, and anti-MAP2 antibodies	Optogenetic stimulation of hDPSCs increased their viability and proliferation. Stimulated hDPSCs exhibited a neuron-like morphology with elongated processes (fibroblast-like) and growth cones. The stimulation also increased the expression of neural markers. The stimulation protocol showed no cytotoxic effects on stem cells.
Michot et al., (5)	DPSCs (cell culture)	CGRP treatment	- MTT assay: following treatment with CGRP - Automated cell counter after 2% Trypan blue stain. - BrdU assay,—IHC detection for anti–caspase–3 antibodies - Alizarin red stain to detect calcified tissue	CGRP inhibits the metabolic activity of DPSCs as these cells showed CGRP did not affect DPSC transformation into mineralizing cells, as assessed by alizarin red stain and odontoblast markers.
Al-Maswary et al., (31)	- hDPSCs - Neuroblastoma cells (SH-SY5Y) (control). (cell culture)	- Successive application of ATRA and BDNF - ERK1/2 inhibitor	- IHC staining for anti-βIII-tubulin, anti-NF-M, and anti-GFAP antibodies - RT-qPCR, for neuronal genes quantification in SH-SY5Y and hDPSC cultures following induction. - ELISA, for Phospho-ERK/MAPK quantification. - Electrophysiological recordings of Na+ and K+ currents.	hDPSCs were transformed into neuronal-like cells using a successive application of ATRA and BDNF. Cells showed neuronal-like morphology and expressed key neuronal markers, with upregulation of cholinergic markers and downregulation of motor neuron markers. The ATRA→BDNF protocol was more effective in hDPSCs as compared to SH-SY5Y cells.
Irfan et al., (6)	hDPSCs and human BM-MSCs. (cell culture) - C5aR agonist and antagonist - LPS	Treatment of stem cells with:	- IHC assessment of anti-C5a receptor, anti-p-p38, anti-STRO-1, anti-BDNF, and anti-NGF) -ELISA evaluation of BDNF from supernatants or cell lysates	C5aR treatment increases BDNF and NGF expression, while the antagonist reduces their production. Inflammation by LPS increased BDNF and NGF secretion in DPSCs and BM-MSCs, but C5aR antagonist reversed this effect. DPSCs outperform BM-MSCs in neurotrophic factor production, with a fourfold increase in BDNF secretion.
Gancheva et al., (33)	Isolated hDPSCs from extracted human impacted 3rd molars (cell culture)	Reprogramming of DPSCs by OCT4 overexpression combined with neuronal induction (multi-step protocol).	- Lentiviral transduction, - Neural induction protocol: Pre-inducing medium (8 days), N2B27 (7 days), then NSC medium (7 days) - Neurosphere formation assay for sphere counting (≥100 µm). - RNA-sequencing and bioinformatics analysis - Xenotransplantation in chicken embryos, GFP^+^ DPSC introduced into chicken embryos. - IHC and Western blot detection of anti-β-III, anti-NF-M, anti-GFAP, and anti-PLP. - RT-qPCR for OCT4 quantification.	The transcription factor OCT4 has been successfully reprogrammed hDPSCs into neural lineages, resulting in a neuronal-like phenotype and potentiate expression of neurona markers. The reprogrammed DPSC showed morphological changes, limited self-renewal, and enhanced differentiation into neurons. In a developmental avian model, the reprogrammed DPSC could survive in the central nervous system and displayed neuronal-like morphologies.
Wang et al., (4)	- hDPSCs obtained from surgically removed 3rd molars. (cell culture) - Male Sprague-Dawley rats aged 6–8 weeks were used for both, IANX and experimental pulp injury model. (animal study)	- CGRP - LPS (inflammatory injury model) - Trigeminal ganglion conditioned medium (TG-CM) - BIBN4096 (CGRP receptor antagonist)	- RNA-seq analysis for differentially expressed genes. - IHC and RT-qPCR for evaluation of TG-CM, CGRP, BIBN4096, and Ramp1. - Transwell chemotaxis assay for recruitment evaluation - ALP and alizarin red staining to detect mineralization. - SEM and Micro CT for dentin bridges evaluation, - Cell proliferation assay.	CGRP binds to the receptor Ramp1 on DPSCs, enhancing their directional movement toward the injury site. Sensory denervation reduced pulp repair capacity and resulted in outside mineralization. Finally, treatment with exogenous CGRP restored DPSC migration and improved pulp repair in denervated pulp.
Wang et al., (32)	- hDPSCs isolated from healthy extracted 3rd molars - Human umbilical vein endothelial cells (HUVECs) (cell culture)	- M2-Exo - M1-Exos - Osteogenic, neurogenic, and angiogenic induction medium	- Uptake assay (PKH-26 labeling) - Cell proliferation assay for exosome-treated cells - Transwell migration assay for detection of treated cells' chemotaxis - Tube formation assay (*in vitro*) and Matrigel plug assay (*in vivo*) for determination of angiogenesis, - ALP and alizarin red staining. - RT-qPCR.	M2-Exos, derived from M2 macrophages, promote cell proliferation and migration in DPSCs and HUVECs, displaying potential for dentin regeneration, neurogenic potential, and angiogenic effects, increasing mineralized nodule formation and vascular networks. while *in vivo*, Matrigel plugs with M2-Exos showed higher VEGF expression, confirming pro-angiogenic capabilities.

DPSCs, dental pulp stem cells; SP, substance P; CGRP, calcitonin gene-related peptide; β-End, beta-endorphin; Met-Enk, methionine-enkephalin; LPS, lipopolysaccharide; VEGF, vascular endothelial growth factor; NGF, nerve growth factor; BDNF, brain-derived neurotrophic factor; GDNF, glial cell line-derived neurotrophic factor; TNF-α, tumor necrosis factor-alpha; IL-1β, interleukin-1 beta; IL-6, interleukin-6; TGF-β, transforming growth factor-beta; Shh, sonic hedgehog; Hh, hedgehog; RAMP1, receptor activity-modifying protein 1; CALCRL, calcitonin receptor-like receptor; Gαs, G-protein subunit alpha-s; AC, adenylyl cyclase; cAMP, cyclic adenosine monophosphate; ERK/MAPK, extracellular signal-regulated kinase/mitogen-activated protein kinase; MDA, malondialdehyde; SOD, superoxide dismutase; IHC, immunohistochemistry; RIA, radioimmunoassay; ELISA, enzyme-linked immunosorbent assay; RT-qPCR, reverse transcription quantitative polymerase chain reaction; RNA-seq, RNA sequencing; TEM, transmission electron microscopy; SEM, scanning electron microscopy; Micro-CT, micro-computed tomography; CFU, colony-forming unit; MTT, 3-(4,5-dimethylthiazol-2-yl)-2,5-diphenyltetrazolium bromide; BrdU, 5-bromo-2′-deoxyuridine; H&E, hematoxylin and eosin; CX43, connexin 43; C5aR, complement C5a receptor; NGFR, nerve growth factor receptor; NF, neurofilament; GFAP, glial fibrillary acidic protein; MAP2, microtubule-associated protein 2; NeuN, neuronal nuclei protein; NSE, neuron-specific enolase; VGluT1, vesicular glutamate transporter 1; GABA, gamma-aminobutyric acid; TH, tyrosine hydroxylase; PIN1, peptidyl-prolyl cis-trans isomerase NIMA-interacting 1; IGFBP5, insulin-like growth factor binding protein 5; OCT4, octamer-binding transcription factor 4; hDPSCs, human dental pulp stem cells; BM-MSCs, bone marrow mesenchymal stem cells; SCAPs, stem cells from apical papilla; GMSCs, gingival mesenchymal stem cells; SH-SY5Y, human neuroblastoma cell line; HUVECs, human umbilical vein endothelial cells; TG, trigeminal ganglion; DRG, dorsal root ganglion; IANX, inferior alveolar nerve transection; M2-Exos, M2 macrophage-derived exosomes; ATRA, all-trans retinoic acid; RA, retinoic acid; b-FGF, basic fibroblast growth factor; EGF, epidermal growth factor; dbcAMP, dibutyryl cyclic AMP).

### 3.1 Temporal phases of neurogenic inflammation

#### 3.1.1 Immediate response (minutes to 24 h)

**Figure 1:**
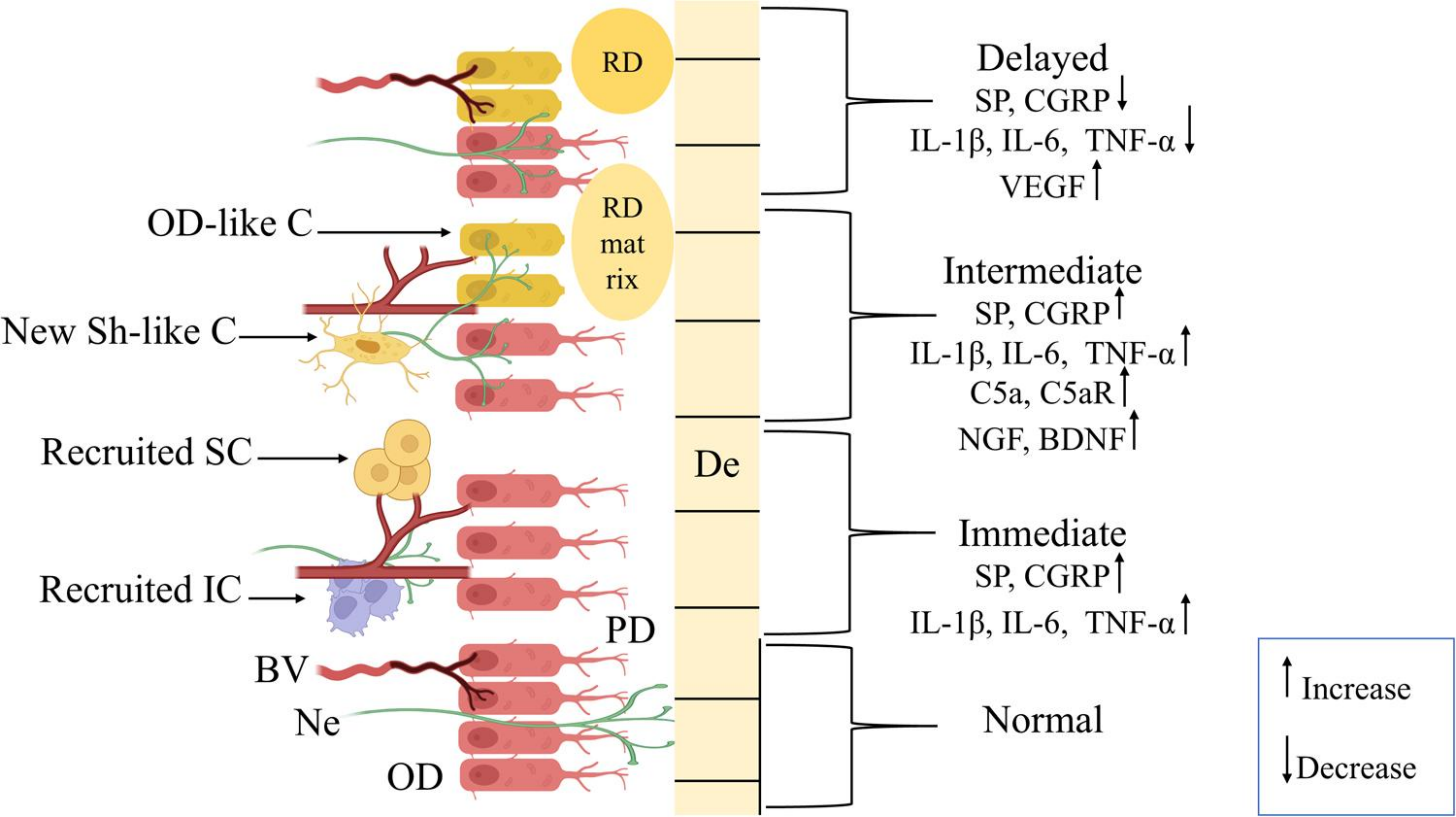
Graphic presentation showing the phases of the neurogenic inflammation process in dental pulp. Normal pulpal tissue exhibits odontoblasts (OD) aligned with intact cellular junctions supplied by normal blood vessels (BV), nerve fibers (Ne) extending between the OD layer through predentine (PD) up to dentin (De). The immediate phase (minutes to 24 h) represents instant pulpal tissue response to noxious stimuli through increased expression of calcitonin gene-related peptide (CGRP) and substance P (SP) neuropeptides, interleukins (IL)-1β, IL-6, and tumor-necrosis factor alpha (TNF-α). And, recruitment of immune cells (IC) and stem cells (SC) with dilation of BV and Ne retraction. In the intermediate phase (24 h to 3 days), a sustained increase in the levels of neuropeptides (CGRP, SP), IL-1β, IL-6, and TNF-α, with elevation in complement 5a and its receptor (C5a, C5aR), nerve growth factors, and brain-derived neurotrophic factor (BDNF). Also, this phase included neural sprouting with differentiation of new Schwann-like cells (New Sh-like C) and odontoblast-like cells (OD-like C), which started to produce tertiary dentin (RD) matrix to initiate the repair process. Finally, the long-term response phase (3 days to several weeks) is characterized by a marked decrease in CGRP, SP, IL-1β, IL-6, and TNF-α levels, an indication of inflammation resolution and neural sprout reduction. Besides, increased expression of vascular endothelial growth factor (VEGF) is associated with angiogenesis, with BV returning to a normal state, and RD formation.

The immediate phase begins minutes to hours after injury or exposure to harmful stimuli, such as bacterial invasion, mechanical trauma, orthodontic forces, or chemical irritants (such as LPS or hydrogen peroxide). Neuronal interaction has been observed early in the pulp reaction to stimuli. As shown in (Figure 1), it comprises the increased release of neuropeptides, including substance P (SP) and calcitonin gene-related peptide (CGRP), from their terminals (2, 15, 16). These neuropeptides are held in large, dense-core vesicles, mostly in C-fibers, and their release is induced by membrane depolarization and increases in intracellular calcium levels (3, 17, 18).

Peptidergic sensory afferents, which account for approximately one-third of all dental pulp neurons, secrete calcitonin gene-related peptide upon stimulation. This neuropeptide binds to receptors on odontoblasts and other pulp cells containing receptor activity-modifying protein 1 (RAMP1) and calcitonin receptor-like receptor (CALCRL). In this way, inflammatory responses can be modulated through the stimulation of G-protein-coupled receptors in these cells. This results in an increase in the intracellular cyclic adenosine monophosphate and activation of adenylyl cyclase. Simultaneously, SP binds to the neurokinin-1 receptors that are found on the different cell types comprising the pulp, fibroblasts, odontoblasts, and endothelial cells. Furthermore, within 24 h of orthodontic force application, there was an obvious rise in SP concentration in the dental pulp. This illustrates the role of this neuropeptide in the initial inflammatory responses due to the application of mechanical orthodontic loading of the teeth (19). These neuropeptides, interacting with their respective receptors, can initiate an inflammatory response cascade such as vasodilation and increased vascular permeability, which result in plasma extravasation and subsequent edema. In conjunction with the mediators that immune cells, for instance, macrophages, neutrophils, and mast cells, are already present, will be recruited. These immune cells amplify the inflammatory processes with cytokines, especially the pro-inflammatory IL-1β, IL-6, and TNF-α. Additionally, SP promotes the degranulation of mast cells, which leads to a release of histamine and other inflammatory components, intensifying the inflammatory response (3). This reflexive relationship between sensory nerves and immune cells perpetuates inflammation of the pulp (2).

Furthermore, odontoblasts are also triggered by neurogenic inflammation. These cells evidenced receptors for neuropeptides, which can stimulate them to release inflammatory mediators and increase their intracellular cAMP levels to modulate cellular function. Although activation of CGRP receptors (CALCRL and RAMP) on odontoblasts by CGRP was reported to inhibit the mineralization process, potentially serving as a protective mechanism to prevent excessive dentin formation as a subsequent increase in pulpal pressure during inflammation (8). This stage also showed the retraction of CGRP fibers from the inflammatory region as indicated in (Figure 1) (15). Also, the odontoblastic processes, along with the sensory nerve fibrils, are evidenced to deeply extend into the dentin tubules. This can affect the pulpal warning system. A histo-immunological study indicated that under typical physiological circumstances, the odontoblast processes are terminated in a form of complex branching network at the dentin-enamel junction (20). This can serve as a “receptor field” to assess the integrity within this region. Therefore, when dentinal tubules were exposed by physiological wear, this can activate a series of actions, including odontoblast processes retraction, possible signaling to the afferent nerve fibers and the sub-odontoblast layer, through odontoblast cell bodies, initiating an inflammatory process (20).

Another role of the neurogenic mediators detected in the early stages of injury or inflammation is the stimulation of DPSCs recruitment and differentiation. It has been documented that CGRP is critical in stimulating DPSCs to polarize and move toward the injury site as a fundamental step in activating the repair process. Autonomous cell movement and collective interaction, termed leader-follower behavior, are crucial for the orderly healing of injured tissues (4, 5). Since DPSCs are of neural crest origin, they possess an intrinsic capacity for neurogenesis and can differentiate into neuron-like cells that harbor neuronal genes (21, 22). Such versatility is important for reconstituting certain parts of neural circuits located in the pulp, such as those involved in nociception and autonomic control, which enable the detection of damaging stimuli and blood flow control (22, 23).

#### 3.1.2 Intermediate phase (24 h–72 h)

The inflammatory process continues during this stage through sustained neuropeptide release and initiation of the tissue repair mechanisms, including new odontoblast-like cells differentiation and production of hard tissue matrix (8). As illustrated in (Figure 1), the levels of SP and CGRP have been reported to be maintained during this stage, as SP expression may peak at 24 h before gradually declining at 72 h after stimulation (18). There can be two major functions of the elevated neuropeptide levels during this stage: First, it is composed of immune cell infiltration and nerve sprouting. Immune cells, such as the dendritic cells, the major antigen-presenting cells, are further evidenced during this stage in the reaction to caries as well as other injuries. They infiltrated the odontoblast layer and extended their processes into the reactionary dentin. Studies have shown that recruitment of dendritic cells occurs parallel to the sprouting of nerve fibers, and this can represent an organized neuroimmune response (24). The complement system, as part of innate immunity, is also involved in neurogenic inflammation. This activation results in the formation of the anaphylatoxins, including C5a, which affect inflammation and the regeneration of nerves. Besides, the expression of C5a receptor (C5aR) has been demonstrated on pulpal fibroblast cells that, upon interaction with C5a, promote the local production of brain-derived neurotrophic factor (BDNF). This may direct axon growth and sprouting toward the injury site (25).

The second role is the transmission of an exaggerated pain sensation as a result of neural sprouting. Nerve fibers are observed to sprout in reactionary dentin against which GAP-43, a marker for neuronal plasticity, is upregulated. This enhancement might be the reason for pain occurring during pulpitis upon caries, as a result of increased nerve fiber density in the affected areas (24). Subsequently, the administration of anti-inflammatory drugs, e.g., hydrocortisone and acetaminophen, by a material (e.g., gel or drug dispersion) was found to influence neuropeptides expression and reduce inflammation in dental pulp. For example, when hydrocortisone (Otosporin) was applied topically after bleaching, inflammation was attenuated. It suppressed the production of SP, CGRP, and facilitated tissue repair (26). Nerve sprouting also occurs as a result of orthodontic forces and is considered to be responsible for the perception of pain after mechanical force is applied (19). Furthermore, CGRP and SP can sensitize nociceptors, which results in mechanical hypersensitivity and spontaneous pain-like behaviors. As in the Calca knockout mice (where the CGRP gene is deleted), there is a significant decrease in spontaneous pain-like behavior but no significant change in mechanical hypersensitivity, which indicates that spontaneous pain sensation is essentially mediated by CGRP (27).

At the intermediate stage, DPSCs recruitment was also demonstrated after CGRP stimulation, and it was reported that DPSCs have a unique receptor named activity-modifying protein 1 (RAMP1) which directly interacts with CGRP, promoting collective attraction of DPSCs to the inflamed area, thus facilitating better repair of the pulp. Additionally, exogenous treatment of CGRP enhanced DPSC mobilization and led to favorable pulp repair quality. Thus, the sensory nerve could be a new target for stem cell-based pulp therapies (4). Besides, a previous study showed that DPSCs can be differentiated into Schwann-like cells using trans-retinoic acid (RA), and essential fibroblast growth factor (b-FGF) (28). This was *in-vitro* study, so it is unknown whether these cells could participate in the formation of new nerve fibers or act as sensory cells by themselves, which is not explained yet by recent studies, and can be a good source for new research. Moreover, DPSCs also exert their immunomodulatory function through the complement system, which was evident when C5aR blocking resulted in a reduction of BDNF levels in DPSCs. This anti-inflammatory activity serves to counterbalance excessive neuroinflammation and to influence neurotrophic secretion. As such, it reinforces the regenerative microenvironment and promotes axonal growth and synaptic connectivity (6).

By the end of this phase, a regeneration process is launched via upregulated NGF expression. These mediators act through p75 and TrkA receptors that odontoblasts and subodontoblasts express, regulating nerve growth, survival, and sensitivity in the response of the host tissues to damage. NGF can also mediate the expression of SP and CGRP in sensory nerves, and so it can affect the inflammatory reaction. In the inflamed pulp, the levels of NGF are upregulated, as well as the ability of DPSC to release BDNF, which might lead to higher susceptibility and more perceived pain during this phase (6, 15).

#### 3.1.3 Long-term response (3 days to weeks or 56 days)

This phase includes events that lead to tissue healing under favorable conditions, primarily influenced by the severity of the injury and the intensity of the immune response. When the outcome of this process is beneficial and the scale tips toward tissue healing, numerous mechanisms work together to achieve this goal, marked by the resolution of inflammation and the initiation of tissue remodeling (16). One of these mechanisms is the upregulation of the vascular endothelial growth factor (VEGF) through the continuous release of CGRP and SP, as demonstrated in (Figure 1). VEGF is essential for the formation of new blood vessels, which secures oxygen supply and nutrients to the pulpal tissue, thereby promoting angiogenesis. This is considered to be a protective mechanism against hypoxia induced by marked inflammation or trauma, preventing tissue necrosis (7).

Yet another mechanism is provided by the reduction in neuropeptides as the SP and CGRP release diminishes in time with abating inflammation. The reduced number and density of nerve sprouts might result in decreasing chronic symptoms over time and help to understand the gradual relief of symptoms (15). Moreover, in response to pain, the body releases beta-endorphins, natural analgesic substances that suppress CGRP release and diminish pain (29). However, in the case of chronic inflammation or repetitive injury, neuropeptide levels can remain elevated, and chronic pain, hypersensitivity, poor wound healing, and possibly even tissue necrosis can result (3, 30). Furthermore, the signaling of CGRP and SP may influence odontoblast activity, eliciting the production of tertiary dentin following injury (7, 8). This role of CGRP in tertiary dentinogenesis was evident in a previous study, which found that the absence of transforming growth factor beta receptor 2 (Tgfbr2) in deficient mice can disrupt TGF-β signaling and impair tertiary dentin formation. at the same time, CGRP immunopositive (CGRP^+^) sensory afferents could compensate for this deficiency by increasing the duration of their axonal sprouting, favoring delayed formation of tertiary dentin, therefore highlighting the protective nature of CGRP during the repair process (30). In addition, sonic hedgehog (Shh) in combination with CGRP signaling, when promoting inflammation after injury, led to proliferation and transformation of DPSCs to odontoblast-like cells for dentin regeneration.

DPSCs have been demonstrated to express multiple neural markers, including glial markers (GFAP, p75, and laminin), and secrete many neurotrophic factors, including BDNF, GDNF, and NGF, through the action of M2 macrophage-derived exosomes (M2-Exos) (31, 32). These exosomes could promote neurogenic differentiation and angiogenesis, which suggests a sophisticated crosstalk between neural and vascular regeneration in the process of pulp repair (32). It has been reported that DPSCs can acquire neurogenic features such as Na^+^ and K^+^ currents and action potentials after neurogenic differentiation. Even though these cells do not entirely recapitulate the function of terminally differentiated neurons, they may help to partly compensate for neuronal signaling in the pulpal healing process (22). In addition, DPSCs might contribute to reducing inflammation and rebuilding the neural and vascular network at the end of this stage by releasing anti-inflammatory cytokines, ensuring the long-lasting function and vitality of the pulp (7).

In the context of the pulpal repair process, many experimental models have been designed to induce DPSCs' neurogenic differentiation. For instance, in an animal model, it has been shown that transplanted DPSCs can engraft to damaged pulp tissue and support the regeneration of neural and vascular structures (33). Accordingly, many *in vitro* techniques are applied to induce neurogenic differentiation in DPSCs that could be used for *in-vivo* in an attempt to produce neurological pulp tissue repair. For example, overexpressing octamer-binding transcription factor 4 (OCT4) could result in the reprogramming of DPSCs into neural lineages implanted in the chicken embryo model. This can induce cellular differentiation to neuronal-like morphology with elevation in the levels of neural markers justifying their role in neural repair (33). Pre-treatment of DPSCs with special molecules (VPA, CHIR99021, Repsox, Forskolin, SP600125, GO6983, Y-27632, Dorsomorphin) also produces transformations of DPSCs into more rounded cell bodies with neurite outgrowth (neural cell morphology). Along with increased expression of neural markers (MAP2, NeuN, and NSE) (21). Furthermore, human DPSCs were differentiated into neuron-like cells after 12 days in all-trans retinoic acid (ATRA) and BDNF treatment, and these cells acquired functional electrophysiological features and neuronal morphology that favor their incorporation into neural networks (31).

Proteins such as peptidyl-prolyl cis-trans isomerase NIMA-interacting 1 (PIN1); insulin-like growth factor binding protein 5 (IGFBP5) increase the neurogenic potential of DPSCs. For example, upregulated PIN1 expression promotes glutamatergic and GABAergic differentiation but suppresses glial differentiation, which may contribute to the recovery of neurons (23). Additionally, IGFBP5 elevates neurogenic-related markers, such as NCAM and nestin, and induces differentiation into neuron-like cells that are beneficial to pulp re-innervation (34). Furthermore, 470 nm, 15 Hz optical stimulation of DPSCs (optogenetic activation) for 5 days enhanced the expression of neural markers and induced neuron-like morphology of the cells. This indicates that optogenetic depolarization drives neurogenic differentiation without notable cytotoxic effects (35). Finally, the ERK/MAPK pathway has been found to be crucial for neurogenic differentiation of DPSCs. Knockdown of ERK1/2 completely inhibited the differentiation of neurons, which was supported by a decrease in the expression of mature neuronal markers (βIII-tubulin, NF-M, GFAP). This suggests that the ERK/MAPK pathway was involved in the sensory cholinergic neuronal transformation of DPSCs (31).

Approaches in the future could enhance the ability of DPSCs to aid in nerve-healing and function-restoration, providing opportunities for treating dental injuries and diseases. This may have implications for new forms of therapy that aid in recovery and stimulate regeneration in injured tissues as well. The application of DPSCs to custom treatments could advance dentistry and the overall well-being of patients. Having outlined the temporal phases, we now discuss the tools to evaluate these processes.

### 3.2 Techniques for assessment of neurogenic inflammation and neurogenic differentiation of dental pulp stem cells

Assessing neurogenic inflammation and differentiation of dental pulp stem cells employs various methodologies ranging from conventional to more sophisticated technologies. Beginning with histological methods, by using non-specific staining, such as hematoxylin and eosin, to assess general histology, inflammation, and necrosis (26). Another more specific staining, including Masson's trichrome staining for collagen and connective tissue analysis (30) and alizarin red staining for calcium deposits and mineralization detection (5). Also, Nissl staining to visualize neuronal cell bodies (23) and trypan blue staining to discriminate between live and dead cells in cell culture, dead cells take the blue stain due to a compromised cell membrane, while live cells repel the dye because of having an intact membrane (5). These stains are assessed mainly under a light microscope. However, subjectivity in scoring means that inflammatory grading (e.g., mild/moderate/severe) or target tissue visualization relies on the examiner's judgment (3). Furthermore, static photos cannot mimic the dynamic processes and real-time progression of inflammation (20).

Immunohistochemistry (IHC), on the other hand, is an antigen-antibody-based reaction in which the primary antibody binds to a special tissue antigen (coupled to an enzyme or fluorophore) to be detected by chromogenic or fluorescent labeling (26). This technique has been extensively applied in dental pulp research for staining tissue sections or isolated culture cells due to its reliability and reproducibility in investigating neurogenic inflammation across various study models (3, 24, 26). It can be utilized to detect cellular receptors (C5aR and NGFR) (25), cytoskeletal proteins (vimentin, α-tubulin, α-actin, and nestin), homeostatic ion transmitters (NaK-ATPase and NHE-1), growth factors (Heng et al.), nerves (CGRP and NF, and glial markers (NaK-ATPase and NHE-1) (20). Advanced live-cell imaging techniques using molecules loaded with calcium and cAMP-sensitive fluorescent dyes. This technique allows simultaneous monitoring of intracellular calcium levels (Ca²^+^ flux) in neurons and cAMP levels in odontoblasts, in real-time signaling fashion. This approach demonstrated that CGRP released from neurons acts as a key mediator in the axon reflex, a neural mechanism that amplifies inflammatory responses in the pulp (8). Nonetheless, IHC is semi-quantitative, lacking precise measurement, which can render comparisons between samples subjective (3, 24). Issues with antibody specificity can result in false positives or negatives due to cross-reactivity or inadequate antibody performance (18). Also, autofluorescence can pose a challenge, as dental pulp tissue may naturally fluoresce, obscuring accurate signals (2). Moreover, photobleaching occurs when fluorophores degrade with prolonged light exposure, diminishing signal intensity along with limited examination time (25).

Another class of immunostaining and molecular detection techniques includes radioimmunoassay (RIA), which is a highly sensitive technique used to quantify neuropeptides such as SP, CGRP, β-Endorphin (β-End), and Methionine-Enkephalin (Met-Enk) in pulp tissue cellular cultures (7, 17). Byers et al. (16) used axonal transport labeling with 3H-proline to trace sensory nerve endings in rat molar dentine. In which autoradiography was employed to detect radio-labeled dentinal tubules, providing insights into the distribution and response of trigeminal nerve endings to tooth wear (attrition). This technique allowed for the quantification of innervated dentinal tubules and the assessment of changes in nerve patterns due to attrition (16). However, radioactive materials necessitate careful handling, disposal, and adherence to safety protocols (17). Despite the high costs and technical demands, its multiplexing capability is limited, typically measuring one analyte at a single time (7). Other types of immunological and molecular techniques, such as in-cell western assays and enzyme-linked immunosorbent essay (ELISA), provide quantitative data on protein secretion, such as BDNF levels, confirming the protein expression of neural markers like nestin and βIII-tubulin (29). However, cross-reactivity of antibodies may result in binding to structurally similar proteins, skewing results (29). In addition to dynamic range, constraints indicate that very high or low analyte concentrations may fall outside the assay's detection limits (28). Another potent laser-based immunological technique for examining physical and chemical characteristics of cells or particles in a fluid solution is flow cytometry. It can be used in the profiling of immune cells during pulp inflammation, such as detection of CD45^+^ (a pan-leukocyte marker), Ly6G^+^ (neutrophils), and Ly6C^+^ (monocytes) (27). Besides, it can be employed in the characterization of stem cells' neural differentiation, e.g., measuring of NeuN, GFAP, Nestin, neural markers of differentiated DPSCs (23).

Other techniques used gene expression to indicate neurogenic differentiation. This can be done by spatial localization of gene expression (*in situ* hybridization), quantifying specific genes [quantitative real-time polymerase chain reaction (RT-qPCR)], or by analyzing the entire transcriptomes (RNA-sequencing) under neural induction conditions. *in situ* hybridization, the labeled probe is applied to tissue sections and allowed to bind (hybridize) to its target sequence to visualize gene activity directly in tissues. Making it invaluable for studies linking genetic changes (e.g., Tgfbr2 deletion) to cellular phenotypes (e.g., defective odontoblasts) (30). In comparison, RNA sequencing is used to uncover how the transcription factor OCT4 reprograms DPSCs into neural lineage cells. By comparing gene expression profiles between OCT4-overexpressing DPSC and controls (33). In addition to gene expression analysis via reverse transcription quantitative polymerase chain reaction (RT- qPCR) to quantify mRNA levels of neurogenic markers such as nestin, GDNF, and SOX 1 (22, 31). However, post-transcriptional discrepancies are present when mRNA levels do not always correspond to protein expression (34) and the fluctuation of reference genes under experimental conditions may result in housekeeping gene variability (4). Another type of gene-based technique is the use of knockout or transgenic methods, which based on the inactivation of a specific gene to identify the missed process within the genetically modified animal. In a study used a knockout mouse model (specifically, Calca^−^/^−^ mice) to assess the role of CGRP in neurogenic inflammation during dental pulp injury. Interestingly, CGRP knockout did not affect mechanical hypersensitivity, suggesting that CGRP is more involved in spontaneous pain rather than evoked mechanically induced pain (27). Although the knockout methodology can specify the exact role of the inactivated gene, it may also affect the overall life of the genetically modified animal, resulting in a series of unexpected consequences (36). Therefore, careful evaluation of the role of these genes should be performed to prevent unsuccessful procedures.

Another class of assessment techniques is cell culture and functional assays, including electrophysiological recordings (patch-clamp), calcium flux assays, and neurite outgrowth in stem cell cultures. These procedures were performed to assess voltage-gated ion channel activity, action potential capacity of differentiated neurons, and length of neurites extended by dorsal root ganglion (DRG) neurons co-cultured with DPSCs, respectively (21, 28). The colony-forming unit (CFU) assay is used to measure the number of colonies produced from single cells under given culture conditions to evaluate the clonogenicity and proliferative capacity of cells, especially stem cells. This was employed in evaluating the effects of CGRP and Shh on DPSCs' proliferation and differentiation (37). While, transwell migration assay is used to assess DPSCs' capacity for migration in response to various chemotactic circumstances. It was used to evaluate how CGRP and M2-Exos affect DPSC migration toward damage sites (4, 32). However, these functional tests have technical difficulties that require skilled operators and stable cell preparations, considered time-consuming and cost-effective for large-scale studies (31). On the other hand, neurosphere formation assays to assess DPSCs neurogenic differentiation through evaluating functional properties (self-renewal) of neural progenitor-like cells by measuring sphere formation in suspension culture, and the expression of neural stem cell markers (e.g., Nestin, SOX1) (23). Since neurospheres are 3D clusters of cells grown in suspension, they are not pure neural stem cells and contain mixed cell populations. This cellular heterogeneity may complicate data interpretation (33). Another functional assay is through the evaluation of mitochondrial activity in live cells by the MTT [3-(4,5-dimethylthiazol-2-yl)-2,5-diphenyltetrazolium bromide] colorimetric test. Metabolically active cells decrease the yellow MTT tetrazolium salt to purple formazan crystals, which are then dissolved, and the absorbance is measured. Such a test is used to quantify metabolically active DPSCs after CGRP treatment (5) and to assess the viability of optogenetically stimulated hDPSCs when no cytotoxicity is observed (35).

For microscopy and imaging techniques, several instruments are used to serve this purpose. Confocal microscopy, which provides high-resolution images, is mainly used for visualizing fluorescently labeled specimens. Transmission electron microscopy (TEM) and scanning electron microscopy (Niyazi et al.) both provide ultra-high-resolution imaging that goes beyond what is possible with light microscopy. TEM is mainly used for ultrastructural analysis of the interior aspect of specimens, as in the observation of myelin-like structures and neurofilaments, and to validate the Schwann cell-like development of DPSCs (28). While SEM is primarily applied to study the surface topography of samples. For instance, SEM was utilized to examine the dentin pulp interface and the deposition of new mineralized dentin matrix following nerve injury (4). Finally, Micro-CT Imaging is a high-resolution, non-destructive 3D imaging approach that allows for the visualization and micrometer-scale analysis of the interior microstructure of mineralized dental tissues. Such as to determine and compare tertiary dentin volume and density after dentinal injury in wild-type mice against Tgfbr2-deficient mice (30).

The limitation in available knowledge is partly due to flaws in the assessment procedure. As noted in the included studies, the most common assessment technique is IHC. This technique is considered invasive and provides only static images of dynamic tissue changes. To date, no technique offers direct visualization of real-time, dynamic living tissue reactions. For this reason, more advanced and noninvasive procedures are needed for direct evaluation of the living sample to gain a better understanding of the complexity of this process. On the other hand, animal models and *in vitro* studies have significantly advanced our understanding of neurogenic inflammation in dental pulp repair, they present critical limitations that necessitate validation through human *in vivo* studies. Among these limitations regarding animal models are the physiological differences (rodent dental pulp anatomy, immune responses, and healing mechanisms differ from humans) (16). And pain assessment challenges [rodent pain behaviors (e.g., grimace scales) are indirect substitutions for human subjective pain experiences, limiting translational relevance] (29). While *in vitro* studies' main limitation is the lack of microenvironment complexity (22).

There are another cutting-edge technique that represent the future of research in this field which have not been shown among the current review electronic search. One of these techniques is spatial transcriptomics, enables researchers to quantify gene expression and identify the specific locations of that expression within a tissue sample (38). Another methodology is the *in vivo* imaging that can provide real-time viewing of the biological processes within a living organism, encompassing a range of technologies such as functional neural imaging for calcium detection to assess neuronal activity using advanced microscopy techniques (39). Organoid models are also an advanced methodology creating tiny, simplified, three-dimensional replicas of organs cultivated from stem cells, replicating essential characteristics of the actual organ's design, cellular variety, and functionality. Among these the neural organoids which have effectively supplemented or replaced animal models to address the distinctive characteristics of human nervous system development (40). Finally, CRISPR screening is a powerful functional genomics tool that uses the CRISPR-Cas9 “gene-editing scissors” to systematically deactivate (or modify) any gene in the genome to underpin its impact on a specific biological process. These advanced technologies offer substantial promise for pinpointing the etiologies and therapeutic targets of neurological disorders (41).

## 4 Conclusions

This narrative review classified the role of neurogenic inflammation within the stage of inflammation into initial, intermediate, and delayed, and each phase is characterized by specific cellular and molecular events. During the immediate phase (minutes to 24 h), neuropeptides are released from sensory nerves, which directly lead to vasodilatation, local inflammation, immune cell recruitment, and early inflammatory signals. The subsequent intermediate phase (24–72 h) is characterized by the persistence of the neuropeptides, nerve sprouting, and the onset of repair processes. Lastly, the long response phase (3 days to weeks) is linked to inflammatory resolution, marked by angiogenesis, reduced neuropeptide levels, and neurogenesis mediated by DPSCs differentiation, and the formation of tertiary dentin. Advanced methods, including IHC, RNA sequencing, electrophysiological studies, and micro-CT imaging, have played a crucial role in the evaluation of these mechanisms. However, limitations in real-time dynamic assessment require the need for non-invasive high-resolution technology to explore the process of pulpal healing. Future studies should focus on the modulation of neurogenic pathways and DPSC-based therapies for enhancing pulp regeneration.
